# Abstracts

**DOI:** 10.1155/S1463924686000329

**Published:** 1986


### Abstracts

### Page 170

### An evaluation of the Waters Pico-Tag system for the amino-acid analysis of food materials

J. A. White *et al.*

An evaluation is described of the Pico-Tag system (Waters Associates) for the rapid, quantitative determination of amino-acids in food materials.

The Pico-Tag method is an integrated technique for pre-column derivatization of amino-acids phenylisothiocyanate (PITC) followed by the separation of PTC-amino-acids by reversed-phase high-performance liquid chromatography. The PTC-amino-acids have a strong ultraviolet absorbance; detection is by measurement of this absorbance at 254 nm.

There was good agreement between results obtained with this analyser and those obtained by the conventional ion-exchange chromatography method of post-column derivatization with ninhydrin.

The Sep-pak method for sample clean-up was investigated for several of the foods analysed.

Both total and free amino-acids may be successfully determined with the Pico-Tag system.

The speed of analysis, repeatability and reproducibility of the chromatography (allowing automated data integration and collection from replicate injections), detection of primary and secondary amino-acids and general reliability of the system make the Pico-Tag method an excellent technique for amino-acid analysis of foods.

---

### Page 178

### A model for cost analysis — application to clinical laboratory test economics using computer facilities

F. R. Hindriks *et al.*

A model for cost analysis in the clinical chemistry laboratory has been developed. All relevant means of production and cost are taken into consideration and allocated to the bearers of the cost, these being a work-station or particular test. The use of this model is discussed in this paper.

It is concluded that the model described is an important tool for laboratory management and of much assistance in discussions concerning staffing, equipment and accommodation requirements. The model is applicable to all laboratories with computer facilities.

### Un modèle pour analyse de frais – application au laboratoire clinique utilisant des ordinateurs

F. R. Hindriks *et al.*

Un modèle pour l'analyse des frais dans le laboratoire chimique clinique a été développé. Tous les moyens de production rélévants et tous les frais ont été pris en considération et attribués au porteurs de ces frais, que ce soit une station de travail ou un test particulier. L'utilisation de ce modèle sera discutée.

Nous pouvons montrer que ce modèle est un outil important dans la gestation du laboratoire et de grande utilité lors de discussions concernant le nombre de personnes employées, le choix des instruments et de la place de laboratoire. Ce modèle s'applique á tous les laboratoires utilisant des ordinateurs.

### Ein Modell für die Kostenanalyse – Anwendung unter Verwendung eines Rechners auf die Oekonomie der klinischen Laboranalytik

F. R. Hindriks *et al.*

Ein Modell für die Kostenanalyse im klinisch-chemischen Labor wurde entwickelt. Alle relevanten Produktionsmittel und Kosten sind berücksichtigt und werden auf die Kostenträger – Arbeitsstation oder Analysenmethode – umgelegt. Die Anwendung dieses Modells wird diskutiert.

Die Schlussfolgerung ist, dass dieses Modell ein wichtiges Werkzeug für das Labormanagment darstellt und für Diskussionen bezüglich Personal, Geräte und Laborausstattung wertvolle Hilfe leistet. Das Modell kann von jedem Labor mit einer Rechnerinstallation eingesetzt werden.

---

### Page 186

### Kinetic-based determinations in continuous-flow analysis

M. D. Luque de Castro and Miguel Valcàrcel

An overview of kinetic methods implemented in continuous segmented and non-segmented flow systems is presented. The characteristics of kinetic determinations and a classification of reaction-rate methods and continuous kinetic-based methods are discussed.

---

### Page 192

### Evaluation of a new semi-automated high-performance liquid chromatography method for glycosylated haemoglobins

A. Carpinelli *et al.*

The authors report data on the evaluation of a new ion-exchange high performance liquid chromatographic system (HLC-723GHb Glycopack-Dyamat), recently introduced by Bio-Rad Laboratories (Segrate, Milano, Italy) to measure the glycosylated haemoglobins in blood samples.

The system operates on haemolysates, which have to be performed separately, by diluting 5 μl of blood with 1 ml of haemolysing reagent.

Each analysis takes about 8 min and the results are printed-out, together with a chromatogram.

Precision and accuracy (comparison with the minicolumn ion-exchange method) were found to be excellent.

The analysis is not affected by the presence of the so-called 'labile fractions', as demonstrated by experiments of incubation with glucose or saline solutions at 37 °C.

Some haemolysates containing abnormal haemoglobins (HbS, HbE, HbJ Paris and Hb Lepore) were also analysed. The effects of the different mutants on the glycated haemoglobins assay are discussed.

The HbF content is also measured by this system, but the accuracy, concerning this particular determination, at the lower concentrations (HbF <2%) is not satisfactory, and needs further improvement.

---

### Page 197

**Serum ionized calcium: evaluation of the Analyte +2 calcium analyser in a clinical chemistry laboratory**
Rosemarie Freaney *et al.*

An evaluation of the Analyte +2 system for the simultaneous measurement of calcium ion activity and pH in serum and whole blood is presented. Correlation of ionized calcium results obtained using the Analyte 2 and a different calcium analyser, the Orion SS 20 system, yielded a co-efficient of 0.986. The precision of ionized calcium determination in serum and in both aqueous and serum-based control materials using the Analyte 2 instrument is reported. Precision was similar whether ionized calcium was uncorrected or corrected to a pH of 7.40. The selectivity of the electrode for calcium was studied by alteration of potassium, magnesium, and sodium. Changes over the physiological range of these ions showed a minimal effect on ionized calcium. The effect of short and long-term storage on ionized calcium and pH was assessed. A reference range for serum ionized calcium in healthy control subjects was established.

---

### Page 202

**Time utilities: programs in MUMPS for calculating intervals of time and for timing events**
T. G. Pellar and A. R. Henderson

The MUMPS language allows the easy manipulation of dates and times. A set of four programs, written in MUMPS, is decribed. The programs (1) calculate time (in days or hours) between events or sequential events; (2) retain files of subjects who are being aimed over a considerable period (for example transplant patient); (3) allow a terminal to be used as a timer; and (4) allow a terminal to be used as an interval timer. These routines have proven to be extremely useful in a hospital department of clinical biochemistry.

---

### Page 207

**Comparative evaluation of a Technicon SMAC2/RA1000 System with an American Monitor Parallel during normal service work**
A. J. Little *et al.*

The choice of a main analyser for many large clinical chemistry laboratories is between the Parallel (American Monitor UK) and a SMAC2/RA1000 combination (Technicon UK). This is a major decision not only because of the large capital outlay, but also it is usually irreversible after installation and acceptance. Thus all available help is enlisted in making the choice, but this is often anecdotal rather than factual and usually not of a comparative nature. In order to provide such information the authors performed a comparative evaluation of these two systems under routine working conditions. Analytical performance, reliability and running costs were investigated over a 15-week period. The study found the SMAC2/RA1000 combination to be more reliable and less imprecise than the Parallel analyser. Conclusion on the relative costing accord to requirements and situations as the two systems employ quite different concepts for sample handling and analysis.

---

### Page 211

**Biochemical laboratory management with a microcomputer**
R. Kowalezyk *et al.*

A Unix multitask microcomputer (Micromega 32:16 or Furtune 32:16) was been interfaced with three analysers Beckman Astra 4, Astra 8 and Greiner G 300 (400 to 600 patients per day and 1500 to 2500 analyses). The independence between the different units of the laboratory has been retained by the division of the software into independent parts. At a given moment, each unit can request: connection to the analyser (background programs which receive and store data), entry of patient reports, data modification, report call-up or printing, plus daily archiving and an activity register. The system's features (response-time, hard-disk capacity, number of input/output) allow it to be expanded to the whole laboratory and linked to the intensive care units.

**Résumé**

R. Kowalezyk *et al.*

Nous avons interfacé un micro-ordinateur multiposte sous UNIX (Micromega 32:16 on Fortune 32/16) avec 3 automates BECKMAN ASTRA 4, ASTRA 8 et GREINER G 300 (400 à 600 dossiers par jour et 1500 à 2500 analyses).

L'autonomie de différentes unités du laboratoire a été préservée par la divison du logiciel en parties indépendantes: chaque unité peut à tout moment demander: la mise en liaison (programmes d'acquisition et de stockage en arrière plan), l'enregistrement, la modification, la visualisation ou l'édition des dossiers ainsi que l'archivage et la comptabilisation des examens.

Les caractéristique du système (vitesse de réponse, capacité disque, nombre d'entrée-asortie) nous permettent de l'étendre à l'ensemble du laboratoire et de procéder à la connexion avec les services de rénimation.

**Management eines biochemischen Labors mit einer Mikrocomputer-Anlage**
R. Kowalezyk *et al.*

Wir haben einen UNIX Multitask-Mikrocomputer (Micromega 32:16 oder Fortune 32:16) mit den drei Analysengeräten BECKMAN ASTRA 4, ASTRA 8 und GREINER 6300 (400–600 Patienten pro Tag und 1500–2500 Analysen) verbunden. Die Unabhängigkeit der verschiedenen Laborgeräte wurde dadurch erhalten, dass die Software in voneinander unabhängige Teile aufgeteilt wurde. Zu jeder Zeit kann jede Einheit folgendes verlangen; Anschluss des Analysengerätes (Programme, die im Hintergrund Daten erfassen und speichern), Eingabe von Patientenberichten, Datenmodifikation, Sichten oder Drucken von Berichten, tägliches Archivieren und ein Aktivitätsregister. Die Eigenschaften des Systems (Reaktionszeit, Festplattenspeicher-Kapazität, Zahl der Ein/Ausgänge) erlauben eine Ausdehnung auf das gesamte Labor une eine Verbindung zu den Intensivstationen.

---

The following were omitted from our last issue; we apologize for their late publication:

**Page 142**

**Automation in urinalysis: sample and data management and quality control**
G. Barbaresi *et al.*

**Automisation dans l'analyse d'urine: gestion d'échantillons et de données et contrôle de qualité**

G. Barbaresi *et al.*

Une méthode pour la gestion quotidienne et le contrôle de qualité d'analyse d'urine est ici décrite. Les échantillons d'urine sont analysés aléatoirement à la réception dans le laboratoire de l'auteur. Utilisant la même séquence aléatoire, les numéros d'identification de patient sont alors entrés dans le mini-ordinateur relié à l'analyseur. A la fin du travail analytique il est possible de vérifier la cohérence des données analytiques et de séparer les échantillons avec des résultats qualitatifs anormaux pour le glucose et les protéines. Les résultats des examens microscopique de sédiment d'urine sont entrés à la main. Un fichier complet des résultats microscopiques et analytiques est enfin transferé à la HDPC (Hospital Data Processing Center) moyennant un floppy disk. Ce HDPD, en utilisant les numéros d'identification, relie les résultats analytiques avec les données du patient (nom, prénom etc.) et imprime une fiche personnelle complète. La cohérence des résultats analytiques est assurée par un programme de contrôle de qualité entre laboratoires, basé sur l'évaluation des résultats d'échantillons d'urine et sur l'analyse de la distribution (au sens analyse combinatoire) des résultats de patients.

**Automatisation bei der Analyse von Urin: Verwaltung der Proben und Daten und Qualitätskontrolle**
G. Barbaresi *et al.*

Es wird ein Vorgehen für die tägliche Durchführung und Qualitäts-kontrolle der Analyse von Urin beschrieben. Urinproben werden in zufälliger Reihenfolge so wie sie im Labor eintreffen analysiert. In der gleichen zufälligen Reihenfolge werden daraufhin dem Minicomputer die Patienten-Identifikationszahlen für die Analysengeräte eingegeben. Nach der analytischen Arbeit ist es möglich, die Zuverlässigkeit der analytischen Resultate zu verifizieren und Proben mit abnormalen qualitativen Resultaten für Glukose unde Protein auszusortieren. Die Resultate der mikroskopischen Prüfung von Urin-Niederschlag werden manuell eingegeben. Ein vollständiger Bericht der mikroskopischen und analytischen Resultate wird schliesslich auf einer Floppy-Disk an das Spital-Rechenzentrum weitergegeben. Im Rechenzentrum werden die Analysendaten mit Hilfe der Identifikationszahlen mit den Patientendaten (Name, Vorname etc.) in Verbindung gebracht und ein individueller Bericht ausgedruckyt.

Die Zuverlässigkeit der Analysenresultate wird durch ein laborinternes Qualitätsprogramm sichergestellt, das auf der Auswertung der Resultate von Kontrollproben und auf der Analyse der Vertilung der Patientenresultate basiert.

### FORTHCOMING PAPERS

The following will be included in 1987's issues:

Element specific detectors.
*S. J. Haswell et al.*

A versatile computerized polarograph, polarographic sample changer and data-acquisition system: applications in electrode mechanism studies.
*H. H. J. L. Ploegmakers et al.*

The systematic error caused by random errors through data reduction.
*K. M. Hangos and L. Leisztner.*

The use of queueing theory for planning automated analytical systems.
*T. L. Pap and L. Leisztner.*

Amperometric determination of nitrogen dioxide in air samples by flow injection and reaction at a gas-liquid interface.
*F. W. Nyasulu and H. A. Mottola.*

